# Forecasting Optimal Solar Energy Supply in Jiangsu Province (China): A Systematic Approach Using Hybrid of Weather and Energy Forecast Models

**DOI:** 10.1155/2014/580606

**Published:** 2014-01-02

**Authors:** Xiuli Zhao, Henry Asante Antwi, Ethel Yiranbon

**Affiliations:** School of Management, Jiangsu University, 301 Xuefu Road, Zhenjiang, Jiangsu 212013, China

## Abstract

The idea of aggregating information is clearly recognizable in the daily lives of all entities whether as individuals or as a group, since time immemorial corporate organizations, governments, and individuals as economic agents aggregate information to formulate decisions. Energy planning represents an investment-decision problem where information needs to be aggregated from credible sources to predict both demand and supply of energy. To do this there are varying methods ranging from the use of portfolio theory to managing risk and maximizing portfolio performance under a variety of unpredictable economic outcomes. The future demand for energy and need to use solar energy in order to avoid future energy crisis in Jiangsu province in China require energy planners in the province to abandon their reliance on traditional, “least-cost,” and stand-alone technology cost estimates and instead evaluate conventional and renewable energy supply on the basis of a hybrid of optimization models in order to ensure effective and reliable supply. Our task in this research is to propose measures towards addressing optimal solar energy forecasting by employing a systematic optimization approach based on a hybrid of weather and energy forecast models. After giving an overview of the sustainable energy issues in China, we have reviewed and classified the various models that existing studies have used to predict the influences of the weather influences and the output of solar energy production units. Further, we evaluate the performance of an exemplary ensemble model which combines the forecast output of two popular statistical prediction methods using a dynamic weighting factor.

## 1. Introduction

China as a growing economy has high demand for electricity and nonrenewable sources of energy have been documented to have low capacity for supporting future social and economic development of China [1]. There is high future demand for energy and a need to explore sustainable alternatives such as the use solar energy in order to avoid future Chinese energy crisis. According to Yang [2] the conceptualization of renewable energy and its sustainability form the foundation of emerging agendas on the future sources and use of energy and energy planning in China as a whole. Solar power, wind power, thermal power, hydropower, and nuclear power are categorized as renewable energy. From sustainability perspective, different sources of power have different life cycle costs, which further contribute to the differences in prioritization of the different renewable power sources [3], although life cycle costs of nuclear power may be lower compared to life cycle costs of solar power and wind power, for example, nuclear plants are exposed to risks of nuclear and radiation emissions that have negative long term impacts on the plants and animals. Solar power has advantage of lower maintenance costs over a long period of time [4].

In China, the alignment of sustainability goals and contribution to socioeconomic development recognize solar power to have the lowest forms of environmental pollution and the lowest level of carbon footprint. For this reason solar power is perceived to be more competitive in China and more research and development have been targeted at improving innovation of solar power in China (at least with the last half of the century) [5]. Apart from its superiority in environmental sustainability goals, solar energy has a higher capacity to satisfy different industrial and domestic needs based on performance measures for achieving energy efficiency, energy saving capabilities, and capacity for reduction of emission technologies. In addition, solar power has higher advantages as a clean energy technology. As Huang et al. [6] have noted planning for energy use within a region or province is a huge investment-decision problem. Currently most of the research works in solar energy issues in China are devoted to issues of demand and investments. Usually demand issues are analyzed by investors using portfolio theory in order to manage risk and maximize portfolio performance under a variety of unpredictable economic outcomes. There is also a modicum of studies in China which encourages those responsible for energy policy formulation and planning to do away with the continuous reliance on traditional, “least-cost” stand-alone technology cost estimates. Instead they should evaluate conventional and renewable energy sources based on the portfolio cost, the cost/risk versus contribution [6].

While the demand for solar energy appears to be relatively high, its supply is not for several reasons in China. Apart from variations, there is also a challenge in the sense that solar energy supply is given attention only in a few provinces in China compared to other forms of energy. For this reason there is the need to develop efficient and dedicated methods of forecasting solar energy as a basis for supporting energy planners and grid operators to efficiently and effectively manage the balance of power between demand and supply of solar energy to overcome any possible instability in and even possible collapses of the sector in the not too distant future [7]. In respect of effective forecasting of solar energy supply, a number of studies exist in the extant literature both at individual and community levels to help cope with the situation.

Even though there is a large deposit of studies which interrogates both practical and scientific optimization ideas, it is still to be determined a straightforward and easy-to-use approach to standardized energy forecasting especially when it comes to solar energy. If the results of different experimental projects are compared, this makes it more difficult to arrive at a simplified and standardized energy forecasting approach because most of these studies analyzed cases that are region specific. Further, there is no constant form of result evaluation across all publications, as different error metrics are applied to measure output quality [8]. Our task in this research is to propose measures towards addressing optimal solar energy forecasting by employing a systematic optimization approach based on a hybrid of weather and energy forecast models. After giving an overview of the sustainable energy issues in China, we have reviewed and classified the various models that existing studies have used to predict the influences of the weather influences and the output of solar energy production units. Further, we describe the process of solar energy forecasting and establish the relevant parameters, settings, and other exogenous influences for selecting the appropriate model against that background. Next, we evaluate the performance of an exemplary ensemble model which combines the forecast output of two popular statistical prediction methods using a dynamic weighting factor [9].

**Figure 1:**
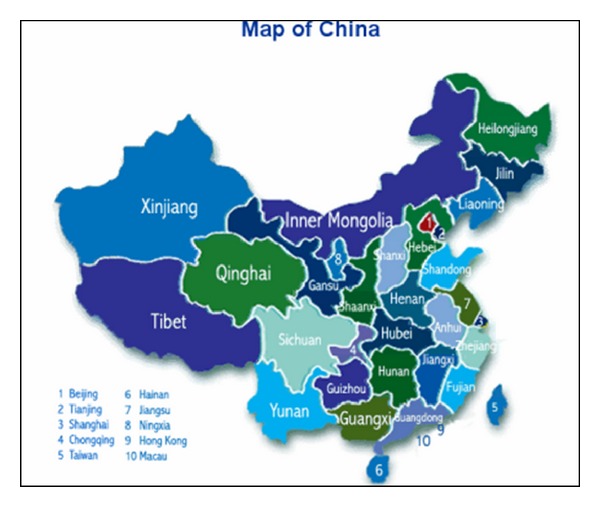
Provincial map of China showing the location of Jiangsu province, adopted from [10].

Following proposal for further studies in existing literature, this study describes essential forecasting ideas in respect of energy planning and discusses their application in the Jiangsu province which is one of the areas in China where solar energy is gaining significant attention (Figure 1 shows the map of China indicating the location of Jiangsu Province). Finally we outline the additional research directions for our future work. Thus, a study on criteria for forecasting methods of solar power could help to determine its sustainability in the long term and capacity to support grid power supply [8]. Further this study is important as it seeks to act as benchmarks for rationale power policy development in global and local contexts and determination of rationale power information systems could be managed [8].

## 2. Renewable Energy Investment in Jiangsu

Currently the Chinese energy demand is largely dependent on coal and crude oil and hydro based energy sources. Recent studies have documented the possibility of increase in demand of power from current 5000–6000 Tonne Watt per hour to 8000–10000 Tonne Watt per hour. The increase in energy demand against decreasing coal-reserves presents possible future challenges and risks of energy crisis or possible economic crisis in China that may arise from energy crisis, aspects that may have more negative economic and financial impacts than was experienced during 1970s Global Financial Crisis that was linked to oil crises; [11] explains that the prospect of exploiting full capacity of solar power cannot be done in haste due to high level of planning and quantification measures that ought to be done and long period that is needed to implement solar power (China National Committee for the Implementation of the UN Convention to Combat Desertification) [11]. This means that the economic and social developments in China may continue to rely on coal and crude oil driven machines due to inability to produce enough alternative sources such as solar energy, wind energy.

Prabhu [12] proposed possibilities of continuity in the use of coal or nonrenewable sources of energy to power national grid system due to high losses that are associated with solar power supply at the point of generation. However this system thinking approach, where high power losses occur at point of generation, could result in nonuse of solar power in large scale or in commercial purposes. But this has been negated by Peidong et al. [13] who proposed that future development of solar power and other renewable sources of energy is important due to lack of sustainability of non-renewable sources of energy from social, economic, and environmental perspectives. This implies that, in China, as emerging higher consumer of electric power, there is, a need for development and innovation of solar power.

Again, according to Qu et al. [14], high energy losses occur at the point of production of solar power which implies that the net energy output (NEO) that is eventually supplied to the National Grid system is lower. Further, as later studies by Fan et al. [15] found, the sellable electricity output from solar farms is very low. The costing of sellable solar power is calculated as a difference between loss of energy output (LEO) and gross energy output (GEO) to give net energy output (NEO) which is documented din literature to be a range between 58% and 47%, from sellable energy percentage of 42% and 53%. For all practical purposes, [15] indicates that solar firms or solar power generators cannot achieve gross energy output (GEO) that is dependent on onsite measurement data and variability of local solar resources. Reference [14] indicate that that differences in GEO arise because different factors are not taken into account when calculating GEO. Some of these factors include

(i) wake effect of solar turbine generator system,
(ii) electric equipment which also consumes part of generated power,
(iii) line losses due to type of wires used and distance that the power is transmitted from production site to consumption point,
(iv) presence of the wake effect of solar-turbine generator systems,
(v) stability or instability of solar speed for turning turbiness,
(vi) changes in the directions of solar leading to variation in the quantity of solar power that is generated,
(vii) changes and variation in weather and climate for instance low or high temperatures and their impacts on air density,
(viii) on-site air density and variation of air density,
(ix) solar farm electricity needs and consumption in the solar farms,
(x) guarantee of power curve of wake effect of solar turbine generator system.

There are fears that solar power may fail to support economic developments the way coal or crude oil power has supported economic developments. However, the high rate of economic development from coal has resulted in increased environmental and resources pressures. For instance, in 1980, in China, acidic rain is documented to have affected over 10% of Chinese land mass. This implies that crude oil and coal as power sources are more pollutant compared to solar power. In addition, solar power does not preclude development of global energy and global power solution. Reference [17] has indicated that the current amount of money that will cost Jiangsu to produce renewable energy is among the highest compared to other options but this cost is actually the initial set-up cost but largely lower when it is spread over its useful life and the consideration is given to other associated social costs. In other words, if renewable energy is compared with other forms of energy such as coals and hydro, on the basis of environmental impact, then using renewable energy is far cheaper in the long term as it helps to bring significant improvement in environment and reduces the susceptibility to other forms of diseases that could have been incurred compared to the high social cost of the rest which are still struggling to get improved technology to make their processing better.

**Figure 2:**
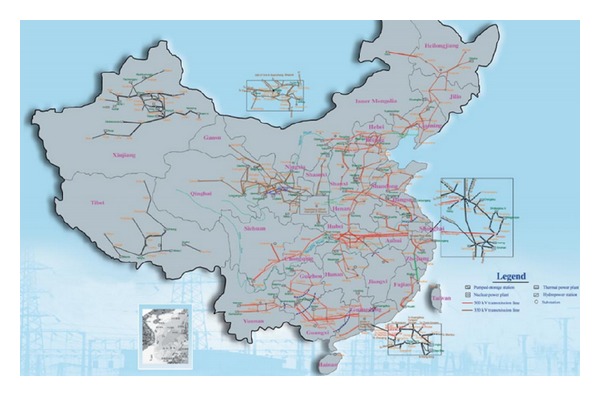
Map of Chinese power grid, adopted from [16].

It is the estimation of Zhong [18] that by the end of the year 2013 the prices of domestic renewable energy technology will decline by averagely less than 5,000 RMB/kW. Since the renewable energy industry all over the world and in China in particular is gradually developing, the trend in prices will continue to decline by about 10% in 2015 and even till about a decade afterwards and will reduce by 10% per decade [18]. It is the confident estimation of [17] that by the year 2030, the prices of renewable energy technology may hit as low as 3,850 RMB/kW or even lower at 3,000 RMB/kW due to the expectation of the emergence of the new efficiency technologies that will make it cheaper to produce and operate the renewable energy technology. On the other hand it is also the case that the unit cost of operating the solar power turbine was going to 0.125 RMB/kWh [19].  Figure 2 shows the map of Chinese power grid.

It has also been estimated by Zhipeng [20] that by the end of the year 2030, the total investment in the solar power if it is looked at from a high scenario perspective will reach about 2.84 trillion RMB, but with an intermediate scenarios the expectation will be that the total investment cost will be 2.13 trillion RMB. On the other hand the low scenario will lead to a total investment cost of approximately 1.57 trillion RMB. From the above analysis and in comparison with other approaches to power generation, Xiaoliang [21] presented evidence to support the claim that not only is renewable energy generation environmentally friendly, thereby having the possibility of reducing the cost of operations in one country as it is in the case of China, but also it has lower operating cost based on all sensitive analyses to the point that even the worst case scenario of costing appears to be more favourable than other methods such as the coal fired energy sources [7]. That notwithstanding it is important for China to target the best case scenario of achieving the lowest cost production and investment since it is within the reach of achievement. Again a recent study [7] categorizes the superiority of solar power energy into three main areas and these are explained below.

## 3. Energy Supply Forecasting Approaches

**Figure 3:**
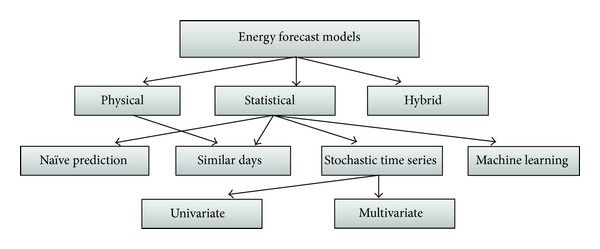
Classification of energy forecasting models, adopted from [8].

Within the existing literature there are several approaches to energy supply prediction; however, the time series remains a common and classical tool for applied energy prediction analysis. According to Alfares and Nazeeruddin [22], the extant literature is inundated with a long and rich history of forecasting electricity load or demand using a range of very sophisticated but high-quality models. However, the necessity for having specialized energy supply forecasting approaches remains a fairly new topic and hence has limited number of studies. The recent interest in energy supply forecasting is largely because of the challenges of grid-connected RES penetrating the distribution systems. Notwithstanding, both energy demand and supply forecasting approaches make use of similar or related techniques. Generally, there are generally two main approaches to energy supply forecasting and these are the weather forecast models and the energy forecast models.  Figure 3 shows the classification of weather forecasting models.

According to Chen et al. [23] the weather forecasting models are computed based on the assumption that energy supply predictions must be done rationally if one wants to get a good forecast. They further explain that a good prediction of the solar energy output is will depend on a good forecast of the solar irradiation. Consequently, using a precise weather forecast model is a functional prerequisite for obtaining or generating a reliable energy output model. This view is shared by Dorvlo et al. [24] who also argue that even though determining the quality of the solar irradiation is orthogonal to the core activities of a grid operator (this is the core work of the meteorological services), having a basic understanding of the underlying principles helps in choosing a specific energy output model. Wu and Chan [25] present series of approaches under the weather forecasting models some of which include the numerical weather prediction, the cloud imagery and statistical models.

Each of these models has different submodels used in enhancing effective prediction. For example, under the numerical weather prediction researchers have used approaches such as the European Center for Medium-Range Weather-Forecasts Model (ECMWF), the Global Forecast System (GFS) from National Centers for Environmental Prediction 4, or the North American Mesoscale Model (NAM). While the numerical weather prediction models above are modern and common method to predict a number of variables by describing the physics and dynamic of the atmosphere, which are then used to derive the relevant weather influences at a specific point of interest, the cloud imagery approaches are relatively different [8].

Using either the satellite data approach or the total sky imagers approach, cloud imagery hinges on the influences of local cloudiness where it is considered to be the most critical factor for the estimation of solar irradiation, especially on days with partial cloudiness where abrupt changes may occur. With the statistical models the treatment of solar radiation forecasting is based on data on historical observation through the use of simple and common time series regression models like ARIMA, artificial neural networks (ANN) or fuzzy-logic models (FL) [8].

According to Reikard [26] after comparing various regression models, ARIMA in logs with time-varying coefficients performs best, due to its ability to capture the diurnal cycle more effectively than other methods [25]. It is further the contention of Wu and Chan [25] that combining ARMA and a time delay neural network (TDNN) is the best while [24] also posit that a hybrid of multilayer perceptron (MLP) and radial basis functions (RBF) which are both ANN-models simpler and precise. On the other hand Mu et al. [27] compares the performance of Adaptative network based fuzzy inference system (ANFIS) against autoregressive models (AR) and ANN. For this reason [8] think that using a statistical model as those above is considered being domain-neutral.

**Figure 4:**
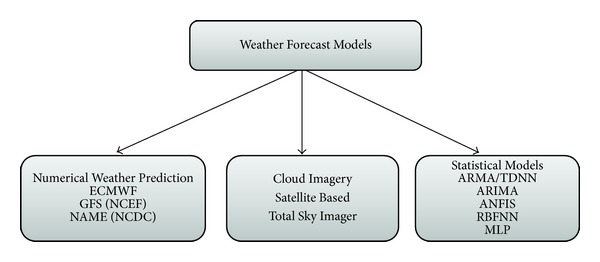
Classification of weather forecasting models, adopted from [8].

On the other hand it is the contention of Cai et al. [28] that when the outputs have been generated from any of the weather models described above, they must equally be converted into energy forecast models. Currently the main energy forecast models or methods include categories of physical, statistical, and hybrid methods as shown in Figure 4.

According to Cai et al. [28] the physical models embraces all the energy supply forecasting approaches that rely on a renewable power plant's technical ability to convert the meteorological resources collected into electrical power. It considers the external influences derived from NWP, local topography, and atmospheric conditions. They are fitted to become an accurate set which has no need of historical output curves. With the local topography, this becomes suitable for the estimation of future output of planned or recently installed RES units. While some levels of solar energy supply predictions have been made by applying physical models, the most frequent and suitable area for their use is in predicting wind energy supply [28].

When it comes to the statistical models for predicting energy, the work of [29] has explained series of them which are used under different conditions. The first of these is the naive prediction model. In this approach considered as the most straightforward of the statistical models naive guess is made by assuming that next periods' expected energy output will be equal to the observations of the current period *P*_*p*_. The next statistical approach to energy prediction is the similar-days model which is based on the concept of diurnal persistence, improved forecasts. This is computed by selecting related historical days with appropriate similarity of time series measures [30].

These models are the most popular for forecasts loads under situations where the weather awareness plays an insignificant role in relation to how consumption cycle derived from historical data influences forecast load. In case of solar energy forecasts, similar-days models are used in instances where there is no NWP available at all or the prediction error included naturally in the NWP is estimated as too high to provide reliable energy output forecasts (Bolinger et al. [31]).

With the stochastic time series models for energy supply forecasting, it is the contention of [8] that this depends on the total number of parameters that influences the models. There are two groups of main approaches to energy supply forecasting in the model and these are univariate and multivariate models. While the univariate employs exponential smoothing for effective one-period ahead, the multivariate model allows for the integration of exogenous parameters. Finally there is the machine learning model to energy supply forecasting. This approach is the most common approach to forecasting a time series' future values. This is because it appears to be the best alternative to conventional linear forecasting methods.

In the existing literature, the ANN has been applied more successfully in forecasting the rate of energy supply fluctuation. ANN learns to recognize patterns in data using training data sets. For example, the use of neural networks is proposed by Frank et al. [32] due to their examination of the feed-forward (FFNN), the radial basis function (RBFNN) and the recurrent neural network (RNN) for solar power forecasting based on NWP input and historical observation data. A similar approach is described by [31] where a RBFNN is combined with a weather type classification model obtained by a self-organizing map (SOM). In contrast, Tao et al. compute hourly energy forecasts using an adaptive NARX network combined with a clear-sky radiation model, which allows for forecasts without including NWP data and still outperforms nonadapting regression based methods.

## 4. Argument for Hybrid Method

According to Kleindorfer et al. [33] the challenge with adopting a single approach top prediction is often the uncertainty that comes with it. The process of planning and predicting is generally an attempt to make a good guess of an uncertain future based on what is happening today. For this reason the ability to get different predictive tools to validate results is essential in effective planning that reduces the risk exposure. In essence the hybrid models refer to the combination of any two or more of the above methods that have been described. According to Jensen [34] using hybrid approaches to predict energy supply pattern has become a very popular approach because of the fact that it gives the opportunity to take advantage of the strongest points of different stand-alone forecasting techniques [35]. The basic idea of combining models is to use each method's unique features in capturing different patterns in the data. Theoretical and empirical findings from other domains suggest that combining linear and nonlinear models can be an efficient way to improve the forecast accuracy [36] so hybrid models seem to be a promising approach that can potentially outperform nonhybrid models individually.

Awerbuch [37] provides a successful example of how hybrid approaches can be applied thus setting a context as to the extent to which this can be applied within the context of Chinese Jiangsu province where solar energy is gaining some level of appreciation over the last couple of years. Specifically in this study we provide an analysis of the impact of the parameters for the selection of energy models for forecasting solar energy supply including the model combination task. From them we compare the total supply quantity forecasted using two stochastic models belonging to the multivariate class: (A) a simple linear model based on principal component analysis and multivariate regression from the MIRABEL project and (B) a commercial library using the more complex non-linear MARS algorithm.

The forecasts are compared both individually and in combination against a naive, weather-unaware reference model (REF) based on the similar-days method. Similar models have been used within the context of Germany by [8]. It is however uncertain as to whether differences in geographical location identified as an important factor in solar energy prediction will affect the level of success of the application of this model in Jiangsu province in China.

## 5. Materials and Methods

### 5.1. Data and Setting for Experiment

Recent studies into prediction market have led to the introduction of model selection criteria of spatial aggregation. In order to cope with this challenge the study included three different observations of solar energy output curves into our adopted scenario. Firstly a single, disaggregated PV-installation located in central Jiangsu town of Nanjing which is incidentally the provincial capital was chosen and is denoted in our model or scenario as DA. Secondly the scenario also considers the aggregate of all PV-installations available that is measured in the same local distribution system and this has been denoted as DS. Thirdly our model equally has an aggregation of all PV-installations attached to the superior transmission system. This however is denoted as TS. The ability to obtain this information requires some level of cooperation; hence, DA and DS data were provided by a cooperating distribution system operator, while data category TS was obtained from a public website. All-time series have a resolution of 15 minutes and cover June 2012 to June 2013.

The study picked data on weather which included measurements of solar irradiation, air temperature, and wind speed with a resolution of 1 hour from a nearby weather station operated by the meteorological services in the province. This was an appropriate source because it is located within the range of the distribution networks. The reason why only the observed weather data was used is because of the ability to eliminate the NWP prediction error. This further allows the energy model performance itself to be evaluated with a high degree of accuracy. From our source time series, we made use of the historical data for the first eleven months for the purposes of training and the last month for forecast evaluation. In order for our scenario to account for day-ahead and intraday terms, forecast horizons of two, twelve, and twenty-four hours ahead were defined in consistency with earlier works of [8, 37–39]. The time thus started each day at 00:15. The model building horizon was adopted following the computation of the forecast. To do this we added the forecast horizon length to the model horizon. This was to extend the length of the available history in accordance with each of the iterations completed. The effect of this was to simulate the integration of newly arriving observations in the forecasting process, which can then be compared with the forecasted values and used to qualify the forecast model. Therefore, the number of forecasting models required to cover the whole month was three hundred seventy-two for a horizon of two hours, sixty-two (twelve hours) and thirty one (twenty-four hours), respectively. In order to derive the optimal combination ratio between two analyzed algorithms *A* and *B*, the variable perceptual weighting factor *λ* was introduced. The final energy forecast *P*_*t*_^*I*^ is then computed by

(1) $$\begin{array}{c} P_{t}^{\prime }=\lambda P_{At}^{\prime }+\left(1-\lambda \right)P_{Bt}^{\prime }. \end{array}$$

In this equation, *P*_*At*_^*t*^ represents the forecasted value of algorithm *A* and *P*_*Bt*_^*I*^ is the forecasted value from algorithm *B* for a timestamp of *t*.

## 6. Output Evaluation 

A number of different statistical metrics available were used to evaluate the extent to which the values that have been forecasted are of good and realistic quality. According to [37] it is often the case that the root mean square error (RMSE) is desirable when one is measuring or evaluating the criterion for intraday forecasts. The reason is that it is able to address the likelihood of extreme values better than others. With the RMSE returning absolute values, we used a normalized form to enable the comparison of the performance of the models on output curves of different aggregation scales. The normalized root mean square error (nRMSE) is achieved by

(2) $$\begin{array}{c} \text{nRMSE}=\frac{\text{RMSE}}{P_{\max}}*100. \end{array}$$

In this model the *P*_max_ represents the maximum observed power output. It was noted that using the percentage difference between observed and predicted power outputs to forecast with day-ahead horizons or above is more interesting. This is expressed by the *mean absolute percentage error* (MAPE). Following [8], nondaylight hours (values with timestamps before 8 am and after 4 pm) and all resting sero observation values were excluded from error calculation. This also means that the effects of snow coverage or measurement failures were removed completely from the results.

## 7. Results of Experiment 

In Table 1, the results of both the linear (A) and non-linear (B) models have been presented. The table shows that both the linear and nonlinear models clearly outperform the similar-days reference model (REF) individually in most of the analyzed cases in terms of nRMSE. Again the information also shows that generally the nonlinear model gives better results than the linear model and this is an indication of the fact that including the historical values improves the quality of output. Notwithstanding, some constellations can be found where model combinations perform slightly better than individual models. With regard to the impact of the chosen forecast horizon, it is observed that the quality of algorithm B decreases with longer horizons and is almost identical to model A for day-ahead periods while model A provides constant values. This can also be explained by the exponential influence of historical values, which has no effect on model A. It is our suspicion that model A can outperform model B on short-term, and midterm forecasts, which have not been covered by the presented scenario.

It appears rather surprising that all models show the lowest preciseness on the DS aggregate where a better performance was expected than that on the disaggregation DA. The possibly included share of self-consumption units (prosumers) may explain the reduction of correlation values, as those measurements are hidden within the observed output curve, thus requiring an individual treatment (e.g., using combined energy demand and supply models) which needs to be further investigated. It is also noted that all models perform best on the highest aggregate. Even though the observed correlation of weather information at only one location is not a representative influence on that supra-regional level, the effect of weather-awareness seems to be completely neutralized by the impact of high aggregation.

## 8. Conclusion

Our task in this research was to propose measures towards addressing optimal solar energy forecasting by employing a systematic optimization approach based on a hybrid of weather and energy forecast models. After giving an overview of the sustainable energy issues in China, we have reviewed and classified the various models that existing studies have used to predict the influences of the weather influences and the output of solar energy production units. Further, we have established the relevant parameters, settings, and other exogenous influences for selecting the appropriate model against that background.

Finally we have evaluated the performance of an exemplary hybrid model that combines the forecast output of two popular statistical prediction methods using a dynamic weighting factor. The study has shown that effective forecasting of solar energy output requires a two-step approach. Firstly it requires weather, and energy forecast models. It has been observed that with respect to energy forecast, there are possible choices that can be selected amongst statistical, physical, and hybrid models. The research has further established that selecting an appropriate model is dependent on various conditions, hence, the forecasting step can be considered as an iterative process. From the effectiveness of the hybrid approach as has been found in the analysis of data, it is evident that a hybrid model that combines both weather and energy forecasting models appears to offer additional optimization option.

This is especially in the case where there is no model found to individually outperform in all given situations. This has been demonstrated in the analysis against the parameters of forecast horizon and spatial aggregation. Notwithstanding we propose that future studies must focus on examining the extent to which complex hybrid forms of energy and weather forecasting models can lead to more efficient predictions. In effect combining many weather forecasting approaches against single energy forecasting approach and vice versa is good enough to start future enquiry into finding the most efficient way of predicting energy supply. The dynamics of solar energy supply in Jiangsu province in China still makes it an interesting field to explore any future complex application of hybrid solar energy prediction models. This may provide some opportunities to develop global standards for forecasting solar energy with more efficient and reliable outcomes.

## Figures and Tables

**Table 1:** Quality of forecast results using nRMSE evaluation metric.

REF	*λ*A	λB	DA 2	DA 12	DA 24	DS 2	DS 12	DS 24	TS 2	TS 12	TS 24
100%	—	—	17,64	18,79	12,99	17,27	20,24	24,96	9,65	10,67	13,33
0	100%	0%	13,66	13,66	13,66	14,68	14,68	14,68	11,80	11,80	11,80
0	70%	10%	13,98	14,28	13,97	13,79	14,39	14,08	9,48	10,14	11,10
0	40%	40%	13,04	12,47	12,89	13,11	14,70	14,39	8,16	9,58	11,30
0	10%	70%	11,84	12,05	13,12	12,29	14,82	15,55	8,73	9,29	11,45
0	0%	100%	11,16	12,54	13,47	12,35	13,88	14,25	8,73	10,51	11,47
